# [Corrigendum] MicroRNA‑378 enhances migration and invasion in cervical cancer by directly targeting autophagy‑related protein 12

**DOI:** 10.3892/mmr.2024.13366

**Published:** 2024-10-17

**Authors:** Dongmei Tan, Chao Zhou, Sai Han, Xuetao Hou, Shufang Kang, Youzhong Zhang

Mol Med Rep 17: 6319–6326, 2018; DOI: 10.3892/mmr.2018.8645

Subsequently to the publication of the above paper, an interested reader drew to the authors’ attention that, for the cell migration and invasion assay experiments shown in Fig. 2 on p. 6322, the ‘HeLa/miR-378 inhibitor’ panels in Fig. 2B and C appeared to contain overlapping sections of data, such that these data panels were apparently derived from the same original source to show the results of purportedly different experiments.

The authors have re-examined their original data, and realize that Fig. 2C was inadvertently assembled incorrectly. The revised version of Fig. 2, now containing the correct data for the ‘HeLa/miR-378 inhibitor’ experiment in Fig. 2C, is shown on the next page. Note that this error did not adversely affect either the results or the overall conclusions reported in this study. All the authors agree with the publication of this corrigendum, and are grateful to the Editor of *Molecular Medicine Reports* for allowing them the opportunity to publish this. They also wish to apologize to the readership of the Journal for any inconvenience caused.

## Figures and Tables

**Figure 2.**
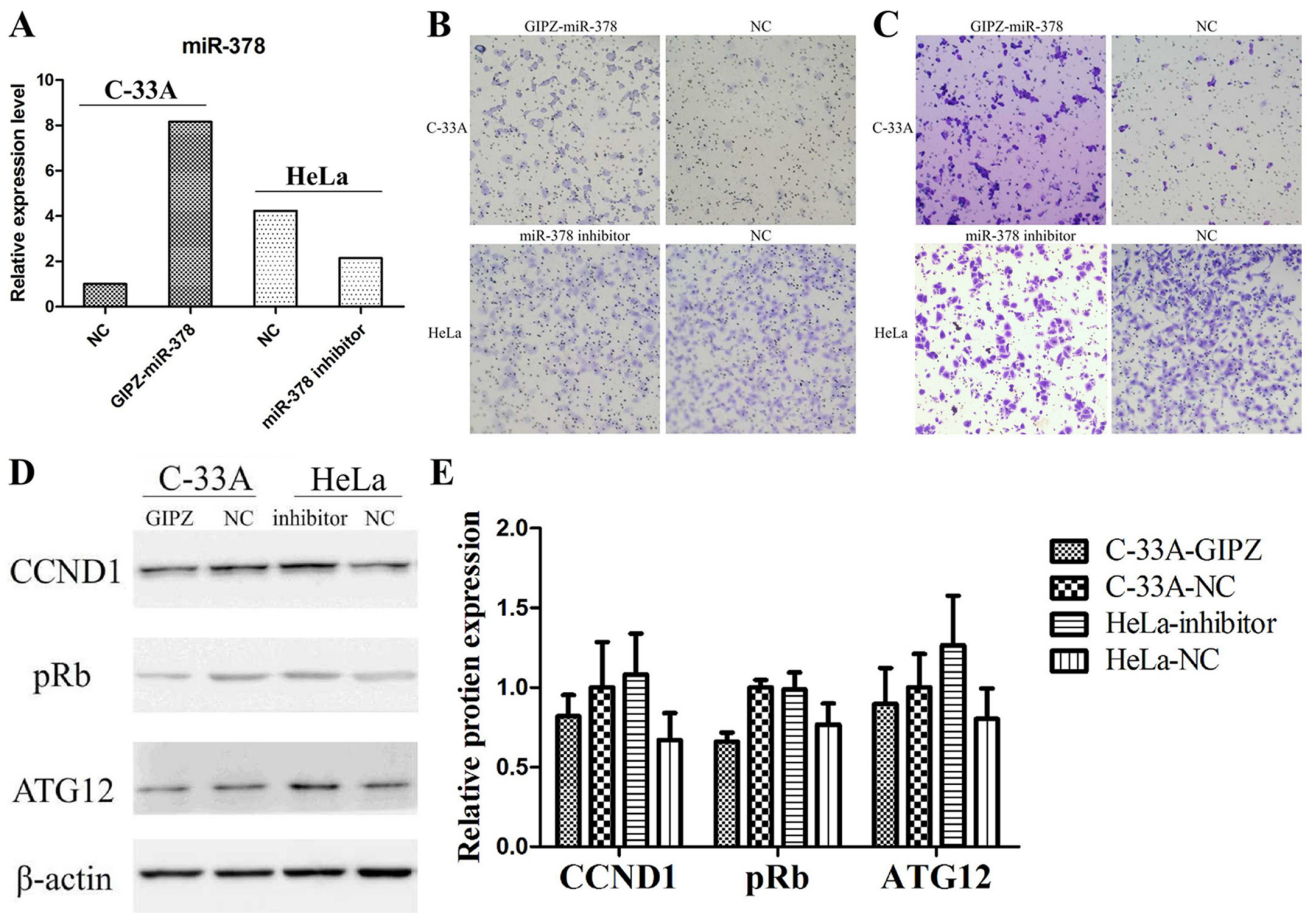
Overexpression of miR-378 promoted cell migration and invasion of cervical cancer. (A) Relative expression of miR-378 was determined by reverse transcription-quantitative polymerase chain reaction. (B) Overexpression in C-33A cells promoted cell migration and knockdown of miR-378 in HeLa cells inhibited cell migration (magnification, ×100). (C) Overexpression in C-33A cells promoted cell invasion and knockdown of miR-378 in HeLa cells inhibited cell invasion (magnification, ×100). (D) Effects of miR-378 on the protein expression of ATG12 were determined by western blot analysis in C-33A cells transfected with GIPZ-miR-378 and HeLa cells transfected with miR-378 inhibitor. (E) Quantitative analysis of the western blot results in Fig. 2D. Each bar represents the grey area of each band and the statistical significance was analyzed from three independent replicates. The data are presented as the mean ± standard deviation. *P<0.05. ATG12, autophagy-related protein 12; CCND1, cyclin D1; miR, microRNA; NC, negative control; pRb, retinoblastoma.

